# AI-Driven Advances in Low-Dose Imaging and Enhancement—A Review

**DOI:** 10.3390/diagnostics15060689

**Published:** 2025-03-11

**Authors:** Aanuoluwapo Clement David-Olawade, David B. Olawade, Laura Vanderbloemen, Oluwayomi B. Rotifa, Sandra Chinaza Fidelis, Eghosasere Egbon, Akwaowo Owoidighe Akpan, Sola Adeleke, Aruni Ghose, Stergios Boussios

**Affiliations:** 1 Endoscopy Unit, Glenfield Hospital, University Hospitals of Leicester, NHS Trust, Leicester LE3 9QP, UK; aanuclement23@gmail.com; 2 Department of Allied and Public Health, School of Health, Sport and Bioscience, University of East London, London E16 2RD, UK; 3 Department of Research and Innovation, Medway NHS Foundation Trust, Gillingham ME7 5NY, UK; stergiosboussios@gmail.com; 4 Department of Public Health, York St. John University, London E14 2BA, UK; 5 Department of Primary Care and Public Health, Imperial College London, London SW7 2AZ, UK; l.vanderbloemen@uel.ac.uk; 6 School of Health, Sport and Bioscience, University of East London, London E16 2RD, UK; 7 Department of Radiology, Afe Babalola University MultiSystem Hospital, Ado-Ekiti 360102, Ekiti State, Nigeria; rotifaoluwayomi@gmail.com; 8 School of Nursing and Midwifery, University of Central Lancashire, Preston Campus, Preston PR1 2HE, UK; sandrachinazafidelis@yahoo.com; 9 Department of Tissue Engineering and Regenerative Medicine, Faculty of Life Science Engineering, FH Technikum, 1200 Vienna, Austria; eghosaseregabriel@gmail.com; 10 Department of Nursing Science, Ahmadu Bello University, Zaria 810211, Kaduna, Nigeria; akwopan@gmail.com; 11 Guy’s Cancer Centre, Guy’s and St. Thomas’ NHS Foundation Trust, London SE1 9RT, UK; olusola.adeleke@kcl.ac.uk; 12 School of Cancer & Pharmaceutical Sciences, King’s College London, Strand, London WC2R 2LS, UK; 13 Department of Medical Oncology, Medway NHS Foundation Trust, Gillingham ME7 5NY, UK; arunighose1@gmail.com; 14 United Kingdom and Ireland Global Cancer Network, Manchester M20 4BX, UK; 15 Faculty of Medicine, Health, and Social Care, Canterbury Christ Church University, Canterbury CT1 1QU, UK; 16 Kent Medway Medical School, University of Kent, Canterbury CT2 7NZ, UK; 17 AELIA Organization, 57001 Thessaloniki, Greece

**Keywords:** radiology, artificial intelligence, low-dose imaging, radiation safety, CT scans, deep learning

## Abstract

The widespread use of medical imaging techniques such as X-rays and computed tomography (CT) has raised significant concerns regarding ionizing radiation exposure, particularly among vulnerable populations requiring frequent imaging. Achieving a balance between high-quality diagnostic imaging and minimizing radiation exposure remains a fundamental challenge in radiology. Artificial intelligence (AI) has emerged as a transformative solution, enabling low-dose imaging protocols that enhance image quality while significantly reducing radiation doses. This review explores the role of AI-assisted low-dose imaging, particularly in CT, X-ray, and magnetic resonance imaging (MRI), highlighting advancements in deep learning models, convolutional neural networks (CNNs), and other AI-based approaches. These technologies have demonstrated substantial improvements in noise reduction, artifact removal, and real-time optimization of imaging parameters, thereby enhancing diagnostic accuracy while mitigating radiation risks. Additionally, AI has contributed to improved radiology workflow efficiency and cost reduction by minimizing the need for repeat scans. The review also discusses emerging directions in AI-driven medical imaging, including hybrid AI systems that integrate post-processing with real-time data acquisition, personalized imaging protocols tailored to patient characteristics, and the expansion of AI applications to fluoroscopy and positron emission tomography (PET). However, challenges such as model generalizability, regulatory constraints, ethical considerations, and computational requirements must be addressed to facilitate broader clinical adoption. AI-driven low-dose imaging has the potential to revolutionize radiology by enhancing patient safety, optimizing imaging quality, and improving healthcare efficiency, paving the way for a more advanced and sustainable future in medical imaging.

## 1. Introduction

Medical imaging has revolutionized modern healthcare by providing non-invasive methods for diagnosing various diseases and abnormalities [1]. Techniques such as X-rays, computed tomography (CT), and nuclear medicine scans allow clinicians to visualize internal structures with a level of detail that was previously unattainable, leading to earlier and more accurate diagnoses [2]. These imaging modalities are essential for detecting and monitoring conditions such as cancer, cardiovascular disease, trauma, and neurological disorders [3]. By guiding treatment decisions and evaluating therapeutic responses, medical imaging has become indispensable in clinical practice [4]. Additionally, recent advancements in radiomics have enhanced the integration of imaging data with other biomolecular insights, uncovering relationships between image-derived features and clinical outcomes [5].

Despite these advancements, medical imaging techniques that rely on ionizing radiation, such as X-rays and CT scans, pose a significant challenge due to the risks associated with radiation exposure [6]. Ionizing radiation has sufficient energy to damage DNA within cells, potentially leading to mutations and an increased risk of malignancies [7]. This risk is particularly concerning for populations that require frequent imaging, such as cancer patients, individuals with chronic illnesses, and pediatric patients [8]. Studies have reported that CT scans alone contribute nearly 50% of the total medical radiation exposure in the general population, despite accounting for only 17% of imaging procedures [9]. Furthermore, excessive and cumulative radiation exposure has been linked to an increased risk of cancers, including leukemia, breast cancer, and thyroid cancer [10].

To mitigate these risks, efforts have been made to reduce radiation exposure in medical imaging. The “As Low As Reasonably Achievable” (ALARA) principle serves as the guiding standard in radiology, emphasizing the need to minimize radiation doses while still obtaining clinically useful images [11]. However, implementing low-dose protocols remains challenging, particularly in CT imaging, where high-resolution images are essential for accurate diagnoses [12]. For instance, a typical abdominal CT scan may expose a patient to a radiation dose of approximately 10 millisieverts (mSv), which is equivalent to about 500 chest X-rays [13,14,15]. This underscores the need for effective protocols that can reduce radiation exposure, especially for patients who require repeated imaging over their lifetime.

Several low-dose imaging techniques have been developed to reduce radiation exposure by adjusting scanning parameters such as tube current, tube voltage, and pitch settings in CT imaging [16]. However, these modifications often introduce trade-offs [17]. Studies have shown that lowering radiation doses typically results in increased image noise, producing grainy or blurry images that obscure critical anatomical details [18]. Such image degradation can compromise diagnostic accuracy, leading to potential misdiagnoses and increasing the likelihood of repeat imaging, which paradoxically raises cumulative radiation exposure [19]. The challenge remains in striking a balance between reducing radiation doses and preserving the high quality of images necessary for accurate clinical decision-making.

**Figure 1:**
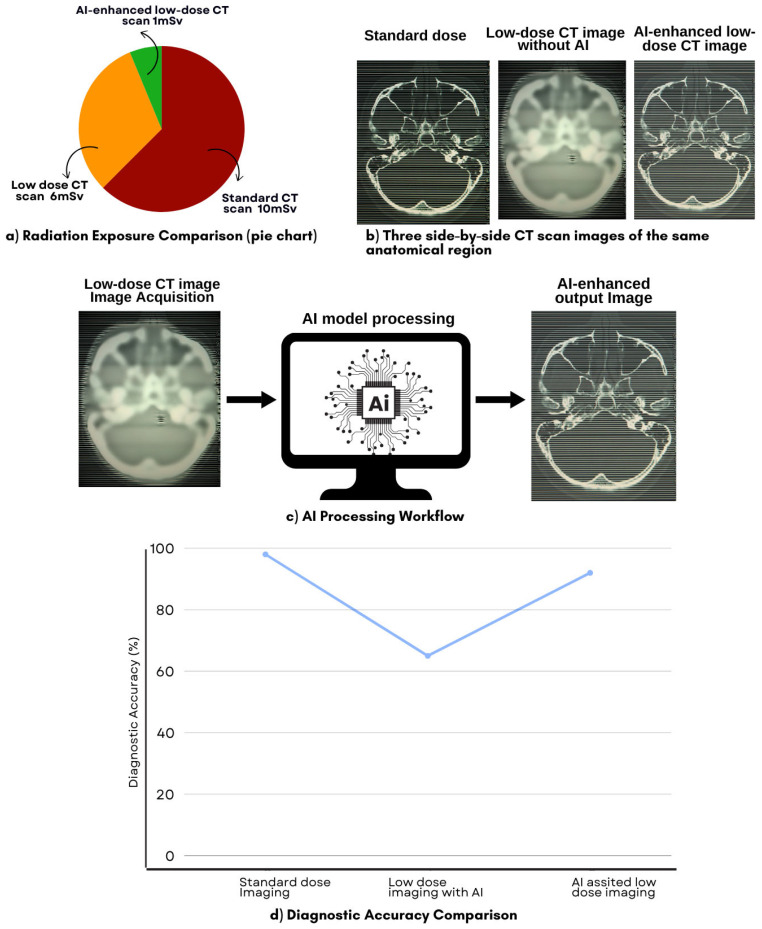
Comparison of radiation exposure, image quality, AI processing workflow, and diagnostic accuracy in standard vs. AI-assisted low-dose CT (LDCT) scanning.

Artificial intelligence (AI) has emerged as a transformative solution to this challenge by addressing the inherent trade-off between dose reduction and image quality [20]. Deep learning (DL) algorithms, trained on large imaging datasets, can reconstruct high-quality images from low-dose scans, effectively reducing noise and correcting artifacts [21]. AI-enhanced imaging techniques improve resolution and contrast, allowing clinicians to maintain diagnostic accuracy even at significantly lower radiation doses [22]. Studies have demonstrated that AI-driven image reconstruction methods can generate low-dose CT (LDCT) images that are nearly indistinguishable from those obtained using standard-dose protocols, thereby enabling safer and more effective imaging practices [23,24]. Figure 1 illustrates the effectiveness of AI-assisted LDCT imaging in reducing radiation exposure, enhancing image quality, and maintaining diagnostic accuracy.

While previous studies have explored low-dose imaging protocols, many have noted that reduced radiation doses often lead to poorer image quality, which can affect clinical decision-making and patient outcomes [25,26,27]. Thus, balancing the need for radiation safety with the necessity of high-quality diagnostic imaging remains a critical issue. AI presents a promising avenue for overcoming these challenges by optimizing image quality while allowing for significant reductions in radiation exposure [28]. This review aims to investigate the effectiveness of AI-assisted low-dose imaging protocols in minimizing radiation exposure without compromising diagnostic accuracy, with a particular focus on their application in CT and X-ray imaging.

Figure 1 presents a comprehensive comparison of radiation dose reduction, image quality enhancements, AI processing workflow, and diagnostic accuracy in AI-assisted LDCT imaging, including (a) a pie chart illustrating the relative radiation exposure for standard CT, LDCT without AI, and AI-assisted LDCT; (b) three CT images of the same anatomical region displayed side by side: a standard dose image, a low-dose image without AI (showing increased noise and reduced clarity), and an AI-enhanced low-dose image (demonstrating improved quality); (c) a simplified AI processing workflow that includes low-dose image acquisition, AI model processing, and enhanced image output; (d) a dot plot comparing diagnostic accuracy rates for standard dose imaging, low-dose imaging without AI, and AI-assisted low-dose imaging, illustrating that AI-enhanced scans maintain diagnostic accuracy while reducing radiation exposure.

## 2. Methods

This study follows the PRISMA (Preferred Reporting Items for Systematic Reviews and Meta-Analyses) guidelines to ensure that a structured and transparent approach to the literature review is followed. The review focuses on AI-assisted low-dose imaging protocols, highlighting their role in reducing radiation exposure while maintaining diagnostic accuracy in medical imaging.

### 2.1. Search Strategy

A systematic search was conducted in the following electronic databases: PubMed, IEEE Xplore, Scopus, and Web of Science. The search covered articles published between January 2010 and December 2023 to ensure an up-to-date analysis of AI applications in radiology. The search terms used included the following: “AI in medical imaging”, “low-dose imaging and AI”, “artificial intelligence in radiology”, “CT dose reduction AI”, “low-dose X-ray image enhancement”, “machine learning in MRI imaging”, “deep learning for noise reduction in imaging”. Boolean operators such as AND/OR were used to refine the search queries and ensure relevant studies were captured.

### 2.2. Inclusion and Exclusion Criteria

Studies were selected based on the following criteria:

#### 2.2.1. Inclusion Criteria

- Peer-reviewed journal articles and conference proceedings discussing AI-assisted low-dose imaging;
- Studies evaluating AI algorithms for radiation dose reduction, image enhancement, or diagnostic accuracy;
- Papers presenting clinical trials, retrospective analyses, or systematic reviews on AI in low-dose imaging;
- Research on AI applications in CT, X-ray, MRI, fluoroscopy, and PET imaging.

#### 2.2.2. Exclusion Criteria

- Studies focusing only on AI in medical imaging without addressing radiation dose reduction;
- Non-English publications;
- Preprints, editorials, or opinion articles without experimental validation;
- Studies lacking quantitative results on AI’s impact on image quality or radiation dose.

### 2.3. Study Selection and Screening

**Figure 2:**
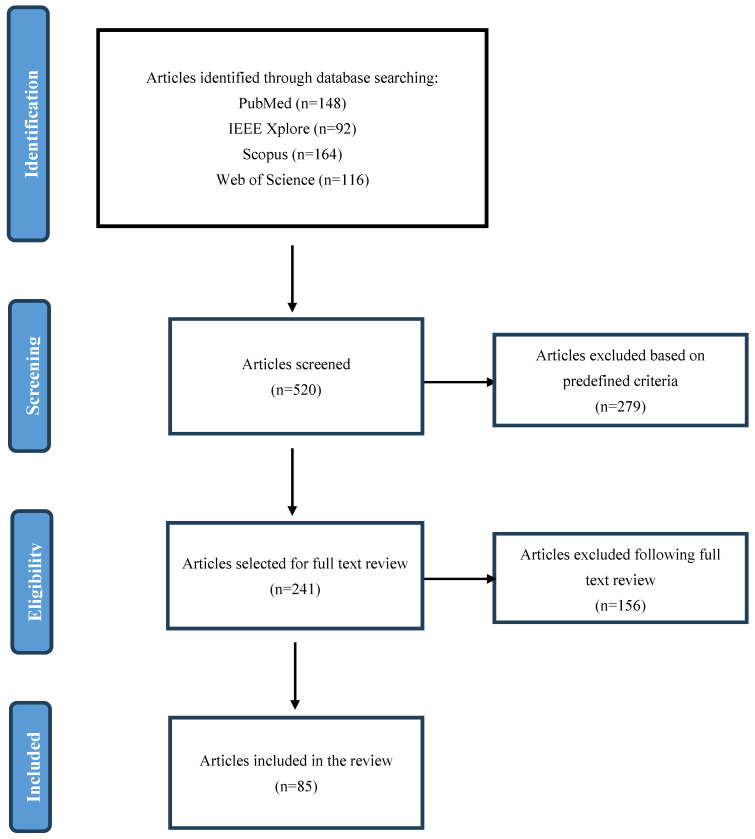
PRISMA flow diagram of the article selection process.

The initial search retrieved 1540 articles across the databases. After removing duplicates (320 articles), the titles and abstracts of 1220 studies were screened for relevance. Of these, 520 full-text articles were reviewed based on the inclusion and exclusion criteria, leading to a final selection of 85 studies for detailed analysis. A PRISMA flow diagram illustrating the systematic search and selection process is included to provide a transparent overview of the methodology (see Figure 2).

### 2.4. Data Extraction and Synthesis

Data were extracted independently by two reviewers and cross-verified to ensure accuracy. Extracted information included the following:

- AI techniques used (e.g., CNNs, GANs, reinforcement learning);
- Medical imaging modality and radiation dose reduction methods;
- Key findings on image quality, diagnostic accuracy, and workflow efficiency.

**Figure 3:**
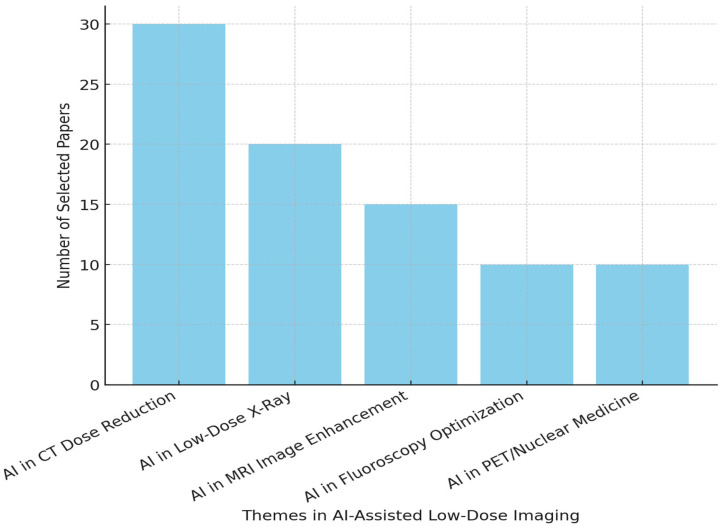
Distribution of selected papers across various themes.

Figure 3 illustrates the distribution of the 85 selected studies across different themes in AI-assisted low-dose imaging. The majority of studies [29] focus on AI applications in CT dose reduction, reflecting the high priority of radiation minimization in computed tomography. Low-dose X-ray imaging follows with 20 studies, emphasizing AI’s role in improving image quality while maintaining low radiation exposure. MRI image enhancement accounts for 15 studies, highlighting AI’s impact in reducing scan times and enhancing resolution. AI applications in fluoroscopy optimization and PET/nuclear medicine each contribute 10 studies, demonstrating emerging research in these modalities.

## 3. Radiation Exposure in Medical Imaging: Risks and Challenges

Medical imaging has become a cornerstone of modern diagnostic medicine, enabling physicians to visualize internal structures and detect disease with remarkable precision [30]. However, imaging modalities that utilize ionizing radiation, such as X-rays and CT, present a growing concern due to the associated radiation exposure risks [29]. In other words, the use of ionizing radiation in diagnostics is invaluable, but it is not without health implications, particularly in cases of repeated or high-dose imaging [31]. As medical imaging technology advances, so too does the frequency of its use, raising questions about the long-term consequences of radiation exposure. While the benefits of imaging, particularly in early disease detection and treatment planning, are substantial, the medical community faces significant challenges in mitigating radiation risks without compromising the diagnostic value of these techniques.

### 3.1. Risks of Ionizing Radiation

According to Akram and Chowdhury, the primary concern with medical imaging procedures that involve ionizing radiation is the biological damage they can cause at the cellular level [32]. Ionizing radiation has enough energy to remove tightly bound electrons from atoms, creating ions [33]. Ionizing radiation can damage the DNA in cells, potentially leading to mutations that, over time, may result in cancer [34]. While a single exposure to ionizing radiation during a medical procedure is generally considered safe for most individuals, a study highlighted that cumulative exposure from repeated imaging studies significantly increases the risk of adverse biological effects [35]. High-dose procedures, such as CT scans, are particularly concerning due to the larger amounts of radiation involved compared to those in standard X-rays [36].

For instance, a full-body CT scan can expose a patient to a radiation dose equivalent to that from several hundred chest X-rays, approximately 10 to 20 millisieverts (mSv) [37]. In contrast, a single chest X-ray exposes the patient to a much lower dose, typically around 0.1 mSv [38]. According to Walsh et al. [39], the cumulative effect of multiple imaging procedures, especially in patients requiring frequent scans, such as those with cancer or chronic illnesses, can lead to significant radiation exposure over time. A systematic review and meta-analysis of early-life ionizing radiation exposure and cancer risks revealed that repeated exposure to ionizing radiation, particularly in younger patients, can increase the risk of radiation-induced cancers [40]. A study demonstrated a measurable increase in the risk of leukemia and brain tumors in children who underwent multiple CT scans [41]. Another study estimated that 1 in every 10,000 children could develop radiation-induced cancer due to repeated imaging [42].

Moreover, findings from a study revealed that pediatric patients are particularly vulnerable to the effects of ionizing radiation due to their developing tissues and longer life expectancy, which provides a larger window for radiation-induced malignancies to manifest [43]. Similarly, another study claimed that certain patient populations, such as those with genetic predispositions to cancer or individuals who require frequent imaging for disease monitoring, are at a heightened risk [44]. These factors reinforce the importance of minimizing radiation exposure whenever possible while still ensuring that medical imaging continues to provide essential diagnostic information.

### 3.2. Efforts to Reduce Exposure

In response to the risks associated with radiation exposure, the medical community has developed several strategies aimed at reducing the amount of radiation used in imaging procedures without compromising the quality of diagnostic images. One of the foundational principles guiding radiation safety is the ALARA principle. The ALARA concept emphasizes that every effort should be made to keep radiation doses as low as possible while still obtaining the necessary diagnostic information [45]. The ALARA concept advocates for the judicious use of imaging procedures, ensuring that the benefits of the procedure outweigh the potential risks of radiation exposure [46]. Radiologists are encouraged to tailor imaging protocols based on individual patient needs, considering factors such as age, size, and the clinical question at hand [47].

To achieve lower radiation doses, healthcare professionals have developed a variety of low-dose imaging protocols, particularly in high-dose modalities like CT [48]. These low-dose imaging protocols often involve adjusting the technical parameters of the imaging equipment to minimize radiation exposure [49]. Additionally, one common approach is to reduce the tube current or voltage during CT scans [50]. By lowering the amount of radiation emitted by the scanner, patients are exposed to lower doses [51]. However, this reduction in tube current or voltage during CT scans typically results in increased noise within the images, which can obscure important details and make it more difficult to accurately interpret the scan [52]. Hence, the trade-off between reduced radiation and image quality is a central challenge in medical imaging, as clinicians must ensure that the diagnostic integrity of the image is not compromised by lower doses.

Consequently, another approach to reducing radiation exposure is increasing the scan pitch in helical CT, which allows for faster scanning with reduced radiation [53]. However, Ahmad et al. [54] argued that this method can introduce artifacts or distortions in the images, which may affect their diagnostic value. To address the limitations of these dose-reduction techniques, there is a development in advanced image reconstruction methods, such as iterative reconstruction [55]. Unlike traditional reconstruction techniques, which rely on filtered back projection, iterative reconstruction algorithms work by processing the image data through multiple refinement cycles [56]. These iterative reconstruction algorithms can reduce noise and improve image quality, allowing radiologists to obtain diagnostically useful images from lower radiation doses [57].

Hence, while these dose-reduction techniques have proven effective, they often come with compromises in image quality, leading to the need for alternative solutions that preserve diagnostic accuracy. This is where AI and machine learning (ML) technologies have shown significant promise. AI-based algorithms, particularly those utilizing DL models, can enhance the quality of low-dose images by reducing noise, correcting artifacts, and improving resolution [58]. By training on large datasets of high- and low-dose images, these models learn to reconstruct high-quality images from low-dose data, making it possible to significantly reduce radiation exposure without sacrificing diagnostic detail [59]. AI’s ability to enhance image quality at lower radiation doses represents a breakthrough in the ongoing effort to make medical imaging safer for patients while maintaining the critical diagnostic value of these technologies.

## 4. Artificial Intelligence in Medical Imaging

AI has emerged as a transformative technology in the medical field, particularly in radiology, where it has the potential to revolutionize image analysis, interpretation, and diagnostic workflows [60]. A study revealed how AI has garnered considerable attention for its ability to automate complex tasks that typically require expert human judgment, while simultaneously augmenting the capabilities of radiologists in the field of medical imaging [61]. Interestingly, the integration of AI into medical imaging not only promises to improve diagnostic accuracy and efficiency but also opens up new possibilities for addressing long-standing challenges such as radiation dose reduction without compromising image quality [62]. AI technologies, including ML and DL models, are increasingly being applied to enhance the quality of low-dose imaging protocols, offering a potential solution to the critical need to minimize radiation exposure while maintaining the diagnostic utility of medical images [21].

### 4.1. Overview of AI in Radiology

AI’s role in radiology has grown substantially in recent years, with the development of sophisticated algorithms that can perform tasks traditionally handled by radiologists [63]. These tasks include image classification, segmentation, detection of abnormalities, and image reconstruction, all of which are crucial for accurate and efficient diagnosis. Among the most impactful AI techniques in radiology are ML and DL, particularly the use of convolutional neural networks (CNNs). A review on efficient neural network techniques reported that CNNs have demonstrated remarkable accuracy in processing large volumes of medical images, learning from the data to identify patterns, detecting abnormalities, and enhancing image quality [64].

**Figure 4:**
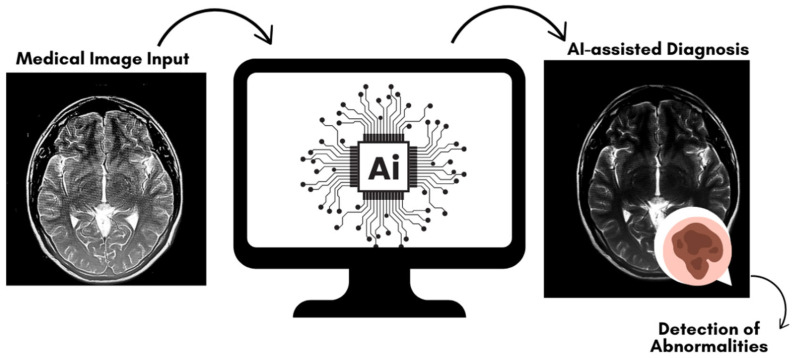
Simplified AI workflow in radiology: from medical image input to AI-assisted diagnosis. The figure illustrates the key stages where AI contributes to radiology, starting from image input, followed by AI processing through classification, segmentation, and image enhancement, leading to AI-assisted diagnosis with improved detection of abnormalities.

One of the major strengths of AI in radiology is its ability to process and analyze vast amounts of image data much more quickly and consistently than human radiologists can, leading to improvements in workflow efficiency [65]. For instance, AI algorithms can be trained to detect lung nodules, brain tumors, or fractures in radiographic images with high sensitivity and specificity, often outperforming human interpretation in terms of speed and accuracy. This capability has sparked considerable interest in applying AI technologies to low-dose imaging, where the challenge lies in the inherent trade-off between reducing radiation exposure and maintaining sufficient image quality for diagnostic purposes [66]. Figure 4 provides a visual that illustrates AI’s contribution to radiology from image input to enhanced diagnosis.

AI’s ability to enhance image reconstruction from noisy or incomplete data makes it an ideal candidate for addressing the limitations of low-dose imaging protocols [67]. Additionally, DL models, such as CNNs, have been developed to learn how to reconstruct high-quality images from low-dose data by compensating for the reduced signal-to-noise ratio that typically results from lower radiation doses. By using large datasets of both full-dose and low-dose images, these models can effectively learn the intricate details of anatomical structures and noise patterns, enabling them to produce images that are diagnostically comparable to those generated by full-dose scans [68]. This application of AI is particularly valuable in modalities like CT and X-ray, where radiation dose reduction is a key priority due to the risks associated with cumulative exposure to ionizing radiation [69].

### 4.2. AI-Assisted Low-Dose Protocols

AI-assisted low-dose imaging protocols leverage advanced algorithms to enhance the quality of images acquired with lower radiation doses, addressing the primary challenge of maintaining diagnostic accuracy while minimizing patient exposure [47]. AI-based solutions have been broadly categorized for low-dose imaging into two main approaches: image post-processing and data acquisition optimization [13,14,15]. The first approach, image post-processing, involves the use of AI models to improve the quality of medical images after they have been acquired [70]. This method focuses on denoising and enhancing low-dose images to compensate for the reduced signal-to-noise ratio that results from lower radiation exposure [71]. AI models, particularly DL-based methods, have shown significant success in this area by reducing the noise and artifacts that typically accompany low-dose scans.

For example, CNNs can be trained to identify and remove noise patterns in CT or X-ray images while preserving important anatomical details [72]. This process allows for significant dose reductions while maintaining image quality at levels comparable to those achieved with standard-dose imaging. In clinical practice, AI-powered post-processing tools have already demonstrated their ability to enhance the diagnostic utility of low-dose scans, reducing the need for repeat imaging and lowering the overall radiation burden on patients [73]. AI is effective in post-processing on LDCT for lung cancer screening, where AI models have been able to denoise images and improve visualization of lung nodules without increasing radiation doses [74]. Similarly, minimizing radiation exposure is particularly critical, and AI-based post-processing algorithms have allowed for the safe use of low-dose protocols while preserving the accuracy required for clinical decision-making [75].

The second approach, data acquisition optimization, integrates AI into the imaging process itself by optimizing scanning parameters in real time to achieve the lowest possible radiation dose while ensuring sufficient image quality. AI-driven systems can analyze patient-specific factors, such as body habitus or anatomical complexity, and adjust the imaging parameters accordingly. This dynamic optimization reduces unnecessary radiation exposure while maintaining high image quality, ensuring that the scan is tailored to the individual needs of the patient [76].

Real-time AI-based optimization has been applied effectively in modalities such as CT, where radiation dose can vary significantly depending on factors such as patient size, tissue density, and clinical indication for the scan. For example, AI can be used to automatically adjust the tube current and voltage during the scanning process, ensuring that the radiation dose is minimized without sacrificing diagnostic clarity [77]. This approach not only reduces the overall radiation burden but also enhances workflow efficiency by reducing the need for manual adjustments and repeat scans. Moreover, AI systems can incorporate feedback from previous scans and continuously improve their performance through ML, further optimizing dose reduction strategies over time [78].

Both image post-processing and data acquisition optimization have shown great promise in clinical applications. There are numerous studies demonstrating the ability of AI algorithms to improve image quality while reducing radiation doses [79]. For instance, a recent study showed that AI-based reconstruction techniques like DL reconstruction algorithms can rapidly reconstruct images to produce desired high-quality CT images at a 30–71% radiation dose reduction [80]. This method, when compared to filtered back projection and hybrid iterative reconstruction, provides better image quality and suggests that AI has the potential to transform low-dose imaging protocols, making them safer and more effective for patients while reducing the risks associated with cumulative radiation exposure.

## 5. AI-Based Image Processing Techniques

AI-assisted low-dose imaging employs various image processing techniques to enhance the quality of medical images while maintaining diagnostic accuracy [81]. These techniques aim to overcome the inherent challenges of reduced radiation doses, such as increased noise, reduced contrast, and image artifacts [82]. By leveraging deep learning-based methods, including convolutional neural networks (CNNs), generative adversarial networks (GANs), and hybrid AI architectures, AI-driven imaging solutions have significantly improved the clarity, resolution, and contrast of low-dose medical images [83]. To validate the effectiveness of these AI-enhanced techniques, standardized evaluation metrics are used to assess image quality, structural similarity, and diagnostic reliability.

### 5.1. Denoising Techniques

Denoising plays a fundamental role in low-dose imaging, as reducing radiation doses often results in a lower signal-to-noise ratio (SNR) [84]. AI-driven denoising techniques aim to suppress noise while preserving anatomical structures to ensure clinical usability [81]. One of the most widely used approaches in denoising is convolutional neural networks (CNNs), which learn noise patterns from large-scale datasets and reconstruct clearer images. CNN-based models have demonstrated an ability to reduce noise by up to 50%, thereby improving image clarity while maintaining diagnostic accuracy.

Generative adversarial networks (GANs) have also been effectively applied in denoising tasks, particularly for low-dose computed tomography (LDCT) images. GAN-based denoising methods, such as Denoising GANs (DnGANs) and Deep Image Prior (DIP), generate high-quality images by learning from full-dose reference scans [85]. These models have achieved structural similarity index (SSIM) scores of over 0.90, indicating near full-dose image quality.

### 5.2. Artifact Reduction Techniques

Artifacts in low-dose imaging often degrade image quality and obscure important clinical information [86]. Traditional iterative reconstruction (IR) techniques have been employed to reduce these artifacts, but they are computationally intensive and can introduce blurring. AI-based approaches, particularly deep learning-enhanced IR methods, have shown significant improvements by optimizing noise suppression and preserving fine anatomical details. Studies have demonstrated that AI-assisted IR improves the contrast-to-noise ratio (CNR) by approximately 30% compared to conventional IR methods.

Autoencoders and U-Net architecture have also been widely used for artifact correction. These deep learning models efficiently detect and remove artifacts in X-ray and CT images, leading to a reduction in mean squared error (MSE) of up to 40% compared to that of traditional reconstruction techniques.

### 5.3. Super-Resolution and Image Enhancement

Super-resolution techniques are employed in AI-driven low-dose imaging to enhance image resolution, making low-dose images comparable to full-dose images [87]. Super-resolution GANs (SRGANs) have shown great potential in generating high-resolution images from low-resolution inputs. These models improve image sharpness and detail, achieving up to fourfold enhancement in resolution for MRI and CT images. Recent advancements in hybrid AI models have combined SRGANs with transformer-based architecture, further improving spatial resolution and texture realism [88]. Such models have been reported to increase the peak signal-to-noise ratio (PSNR) by 4–6 dB over that of conventional upscaling methods, resulting in improved image clarity without introducing excessive noise or artifacts.

### 5.4. Contrast Enhancement Techniques

Contrast enhancement is critical in medical imaging, as it allows for the better differentiation of tissues and the detection of abnormalities [89]. Low-dose imaging often results in reduced contrast, which can compromise the visibility of lesions or subtle pathologies [90]. AI-based contrast enhancement techniques, such as histogram equalization and adaptive contrast enhancement, have been successfully implemented to improve the contrast-to-noise ratio (CNR) [91]. Deep learning approaches using reinforcement learning algorithms have been applied to optimize contrast levels dynamically [92]. These techniques have been shown to improve lesion detectability in positron emission tomography (PET) scans by 15–20% compared to standard contrast adjustment methods. AI-driven contrast optimization also helps in refining brightness levels, ensuring uniform contrast across various tissue densities.

## 6. Evaluation Metrics for AI-Based Low-Dose Imaging Techniques

To quantitatively assess the effectiveness of AI-driven low-dose imaging techniques, various evaluation metrics are used to compare AI-enhanced images with their full-dose counterparts. These metrics evaluate image quality, structural similarity, contrast enhancement, and diagnostic performance. Table 1 presents the most commonly used metrics and their significance in evaluating AI-based low-dose imaging methods.

## 7. Key Applications of AI in Low-Dose Imaging Protocols

The integration of AI into medical imaging has revolutionized several imaging modalities, with a particular focus on reducing radiation exposure while maintaining image quality. AI’s ability to analyze and reconstruct images with minimal noise and artifacts makes it particularly suitable for low-dose imaging protocols. Table 2 provides a concise summary of how different AI techniques are applied across various imaging modalities, their functions, benefits, and an example or study supporting each application.

### 7.1. AI in CT

CT is one of the most widely used imaging modalities due to its ability to provide detailed cross-sectional images of the body. However, CT imaging is associated with relatively high radiation doses, especially when compared to other imaging techniques like X-rays [123]. As a result, CT imaging has been at the forefront of AI-driven dose reduction strategies, with a focus on minimizing radiation exposure while maintaining the diagnostic integrity of the images. AI algorithms, particularly DL models, have demonstrated remarkable potential in improving the quality of LDCT images. Techniques such as deep CNNs and generative adversarial networks (GANs) are being utilized to enhance the quality of images obtained with reduced radiation doses. These models excel at performing image denoising, artifact reduction, and image reconstruction, enabling the acquisition of diagnostically useful images with much lower radiation doses than traditional protocols [47].

For instance, deep CNNs are trained on large datasets of both full-dose and LDCT images, learning the complex patterns in anatomical structures as well as the noise and artifacts introduced by reduced radiation exposure. This enables the model to reconstruct high-quality images from low-dose inputs. GANs, on the other hand, can generate synthetic high-quality images by learning to map low-dose images to their full-dose counterparts, thus preserving the diagnostic information in a fraction of the radiation dose. This approach has been shown to significantly reduce the noise and improve image resolution [27,115].

Numerous studies have demonstrated the clinical utility of AI in LDCT. Researchhas shown that AI-enhanced LDCT images can achieve comparable diagnostic accuracy to those obtained with full-dose scans [124]. For example, in lung cancer screening, where LDCT is frequently used to minimize radiation exposure, AI-based reconstruction methods have successfully maintained the ability to detect small lung nodules, which are critical for early diagnosis. The integration of AI into LDCT has not only reduced radiation risks but has also improved the overall efficiency of the imaging process by decreasing the need for repeat scans due to poor image quality [74].

### 7.2. AI in X-Ray Imaging

X-ray imaging is another modality where AI-assisted protocols have been applied to reduce radiation exposure while maintaining image clarity. Although X-rays expose patients to lower radiation doses than CT scans, the frequent use of this modality in clinical practice, particularly in pediatric and emergency settings, necessitates dose reduction strategies [125]. AI techniques, particularly those involving ML and DL, have been instrumental in improving the quality of low-dose X-ray images by reducing noise, enhancing contrast, and preserving anatomical details that are crucial for diagnosis.

Some of the primary challenges in low-dose X-ray imaging are the increased noise and reduced contrast that result from lowering the radiation dose. AI-based denoising algorithms have been developed to address these issues by learning from large datasets of X-ray images. ML models, trained on both low-dose and full-dose X-ray images, can identify and filter out noise while preserving important anatomical features such as bone structures, soft tissues, and lesions [126].

These models not only enhance image clarity but also maintain the diagnostic value of the images, ensuring that clinicians can make accurate assessments even with reduced radiation exposure. In clinical applications, AI-enhanced low-dose X-rays have been particularly beneficial in pediatric imaging, where minimizing radiation exposure is critical [116]. AI-driven denoising and contrast enhancement have made it possible to safely use lower radiation doses while still achieving the diagnostic detail necessary for conditions such as fractures, infections, or congenital abnormalities. Moreover, in emergency settings where X-rays are often performed rapidly, AI-assisted low-dose protocols have helped improve the speed and accuracy of image interpretation, reducing the need for repeat scans and improving patient throughput [117].

### 7.3. Magnetic Resonance Imaging (MRI) and AI

Although MRI does not involve ionizing radiation, AI-assisted protocols have been explored in MRI to optimize other safety-related factors, such as reducing scan times and minimizing the use of contrast agents. MRI scans are known for their long acquisition times, which can lead to patient discomfort and motion artifacts that degrade image quality [127]. Additionally, some MRI procedures require the use of contrast agents, such as gadolinium, which can pose risks in certain patient populations, particularly those with kidney dysfunction. AI-driven denoising and image reconstruction algorithms have been developed to address these challenges, allowing for faster MRI scans and improved image quality. These AI models, much like those used in CT and X-ray imaging, learn from large datasets of MRI images and can enhance images by reducing noise and correcting for motion artifacts [119]. This not only shortens scan times but also reduces the likelihood of repeat imaging, making the process more efficient for both patients and clinicians. Quantitative data from different surveys showed out that over 80% of patients expressed a positive overall experience with the improved imaging procedures [121].

**Figure 5:**
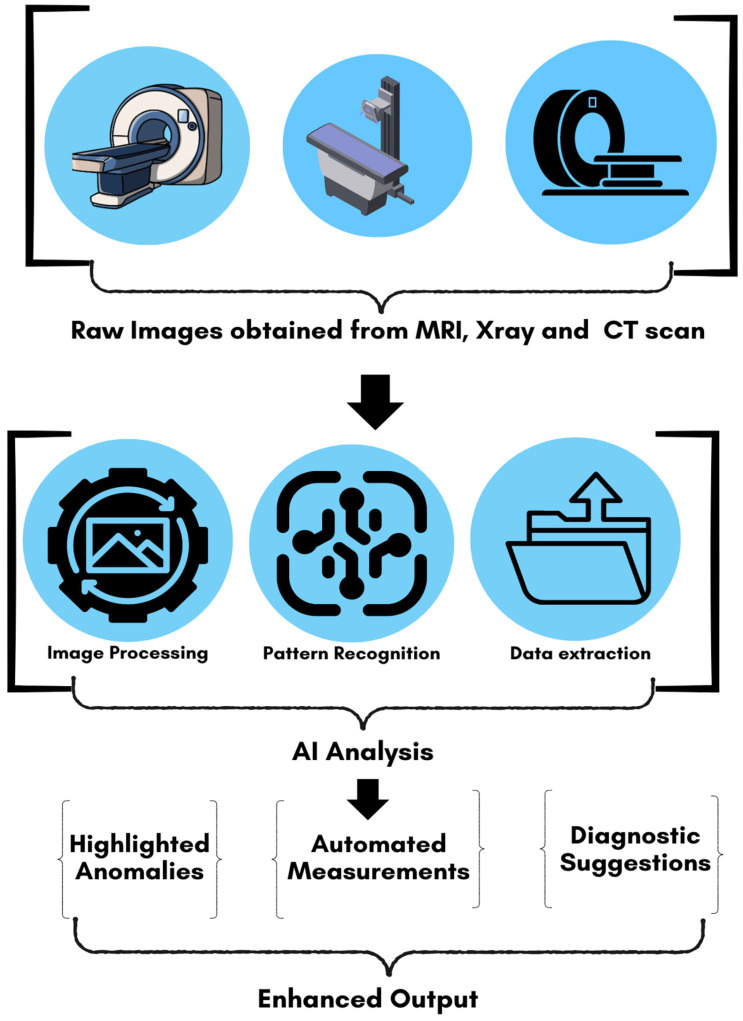
Simplified workflow of AI applications in medical imaging.

AI-based algorithms are also being used to improve the resolution of images acquired with reduced scan times. Traditionally, reducing scan time in MRI leads to a loss of image resolution, but AI algorithms can interpolate and reconstruct high-resolution images from shorter scans [128]. This capability has been particularly valuable in applications such as brain imaging, where high-resolution detail is critical for diagnosing neurological conditions. Additionally, AI-assisted MRI protocols have contributed to safer imaging practices by reducing the need for contrast agents. In some cases, AI-driven reconstruction techniques can enhance the visualization of tissues and structures to the point where contrast agents are no longer necessary, particularly in patients at higher risk for adverse reactions. For example, a study explored the use of AI-based methods to enhance the visibility of tumors or blood vessels in non-contrast MRI scans, providing an alternative to gadolinium-based agents [129]. Figure 5 demonstrates how AI can augment medical imaging across different modalities, potentially improving diagnostic accuracy and efficiency. This diagram illustrates the basic process of applying artificial intelligence to MRI, X-ray, and CT scan images. The workflow consists of three main stages: (1) acquisition of raw images from various imaging modalities, (2) AI-driven analysis incorporating image processing, pattern recognition, and data extraction techniques, and (3) enhanced output providing highlighted anomalies, automated measurements, and diagnostic suggestions.

## 8. Benefits of AI-Assisted Low-Dose Imaging

AI-assisted low-dose imaging has introduced transformative benefits in the field of medical imaging, providing solutions to long-standing challenges such as radiation safety, image quality, and healthcare efficiency. These benefits extend across a wide range of clinical applications and patient populations, making AI a critical tool in improving diagnostic accuracy while reducing the risks associated with imaging procedures.

### 8.1. Reduced Patient Risk

One of the most significant advantages of AI-assisted low-dose imaging protocols is the reduction in radiation exposure, which in turn lowers the risk of radiation-induced harm [130]. This is particularly important for vulnerable populations, such as pediatric patients, cancer patients, and individuals requiring frequent imaging for chronic conditions. These groups are at an elevated risk of radiation-related complications due to their increased exposure over time. For example, pediatric patients are more susceptible to the effects of ionizing radiation due to their developing tissues and longer life expectancy, which increases the likelihood that radiation-induced malignancy could develop later in life. A study conducted on the late effect of radiation therapy in pediatric patients and survivorship found out that although advances in multimodality therapy have led to childhood cancer cure rates of over 80% [131]. However, surgery, chemotherapy, and radiotherapy may lead to debilitating or even fatal long-term effects among childhood survivors beyond those inflicted by the primary disease process [131]. Similarly, cancer patients undergoing routine surveillance imaging often accumulate higher radiation doses, compounding their risk of secondary radiation-induced cancers.

AI-assisted low-dose imaging directly addresses this issue by allowing clinicians to obtain high-quality diagnostic images using significantly reduced radiation doses. According to reports from a systemic review on AI for radiation dose optimization in pediatric radiology, a majority of studies demonstrated that AI could reduce radiation doses by 36–70% without causing a loss of diagnostic information, with three studies included in the systematic review demonstrating that the use of AI could even achieve further radiation dose reductions of up to 95% [132]. By minimizing radiation exposure, AI reduces the cumulative radiation burden on patients, significantly lowering their long-term health risks while still providing the imaging data necessary for effective diagnosis and treatment planning.

### 8.2. Enhanced Image Quality

One of the primary challenges of traditional low-dose imaging protocols has been the degradation of image quality, which can lead to decreased diagnostic accuracy [133]. Lowering the radiation dose typically results in increased noise and reduced contrast, making it difficult for radiologists to identify subtle abnormalities. However, AI-assisted imaging protocols offer a solution to this problem by using advanced algorithms to enhance image quality, even when the radiation dose is reduced. A comparative study on coronavirus disease (COVID-19) CT images and its findings were supportive of AI’s ability to enhance image quality through denoising, artifact removal, and image reconstruction, which is particularly valuable in imaging modalities, where maintaining clear, high-resolution images is essential for accurate diagnosis [134].

AI models, particularly DL algorithms like CNNs, can process low-dose images and remove noise without losing critical anatomical details [135]. This allows radiologists to detect small lesions, fractures, or other abnormalities that might otherwise be obscured in low-dose images. For example, AI-enhanced LDCT scans have been shown to produce images that are diagnostically equivalent to full-dose scans, enabling the detection of lung nodules or the obtention of other critical findings without the need for higher radiation exposure [115]. In addition to noise reduction, AI algorithms can correct for motion artifacts or distortions that occur during imaging, further improving the clarity and accuracy of the images [136]. This ensures that even with lower radiation doses, AI-assisted imaging provides the high-quality images necessary for effective clinical decision-making.

### 8.3. Cost Efficiency

AI-assisted low-dose imaging protocols also contribute to cost efficiency in healthcare by reducing the need for repeat scans and improving overall workflow efficiency [73]. Poor-quality images that result from traditional low-dose imaging protocols often necessitate additional scans to obtain clearer diagnostic information, increasing both the radiation dose to the patient and the overall cost of care. Repeat imaging not only adds to patient exposure but also places additional strain on healthcare resources, leading to increased costs for hospitals, imaging centers, and patients alike. By enhancing image quality at lower radiation doses, AI can significantly reduce the frequency of repeat scans, thus lowering the overall cost of healthcare [137]. AI algorithms optimize imaging protocols in real-time, ensuring that the first scan produces diagnostically useful images, which minimizes the need for additional imaging. This improved efficiency not only enhances patient safety but also reduces operational costs associated with extended imaging sessions, equipment wear, and personnel time. Furthermore, AI-driven imaging solutions can streamline the diagnostic workflow by automating certain aspects of image interpretation. AI algorithms can pre-process and enhance images, allowing radiologists to focus on interpretation rather than manual image correction. This leads to faster diagnostic turnaround times, improves throughput in busy radiology departments, and ultimately enhances patient care. By integrating AI into imaging protocols, healthcare providers can achieve more efficient use of resources, improving both clinical and economic outcomes [138].

## 9. Future Directions

As AI continues to make significant strides in the field of medical imaging, the potential for further advancements in low-dose imaging protocols is vast. The authors of [47] suggested that the next generation of AI-driven imaging systems is expected to push the boundaries of radiation dose reduction while maintaining or even enhancing diagnostic quality. In a study, it was concluded that by focusing on hybrid AI systems, personalized imaging protocols, and the expansion of AI applications to other imaging modalities, the future of AI-assisted medical imaging holds the promise of even safer and more efficient diagnostic techniques [139].

### 9.1. Hybrid AI Systems

One of the most exciting areas of future research in AI-assisted low-dose imaging involves the development of hybrid AI systems that combine both image post-processing and real-time data acquisition optimization [140]. While current AI models typically focus on either post-processing (enhancing images after acquisition) or optimizing scanning parameters during the imaging process [141], future hybrid systems could seamlessly integrate both approaches to maximize the benefits of each. These hybrid systems, as accessed, would enable real-time feedback during the imaging procedure, allowing for the continuous adjustment of scanning parameters based on AI-driven analysis of the patient’s anatomy and the specific imaging requirements [142]. After image acquisition, the system would then apply advanced post-processing algorithms to further enhance image quality, removing noise and artifacts that may have resulted from low-dose scanning [143]. However, this dual approach has the potential to provide even greater reductions in radiation dose while ensuring the highest level of diagnostic accuracy [144]. Moreover, hybrid systems could learn from each imaging session, continually improving their performance over time, leading to increasingly personalized and optimized imaging protocols for each patient [145].

### 9.2. Personalized Imaging Protocols

In 2015, a study reported extensively on another promising direction for future AI research, which is the development of highly personalized imaging protocols. They explored the possibility of this in the field of cancer radiation therapy [146]. Just as practiced in clinical radiology, current imaging protocols are generally standardized, meaning that all patients undergoing a particular imaging study receive roughly the same radiation dose, regardless of their individual characteristics [147,148]. However, another modeling study concluded that AI offers the opportunity to tailor imaging protocols based on patient-specific factors, such as body mass index (BMI), age, medical history, and the specific clinical question being addressed [149].

Personalized imaging protocols driven by AI would allow for more precise adjustments to radiation doses, ensuring that each patient receives the minimum necessary exposure while still achieving diagnostically sufficient images [150]. For instance, it was concluded that incorporating patient-specific body metrics into CT dosimetry could enhance personalized care and radiation safety [150]. Patients with higher BMI may require different imaging parameters than those with lower BMI due to differences in tissue density, and AI could adjust the dose accordingly. Similarly, AI could factor in the patient’s medical history, such as the frequency of previous imaging exams or known radiation sensitivity, to further refine dose settings [22]. By incorporating these individualized factors, AI-driven personalized protocols could not only reduce unnecessary radiation exposure but also improve the overall quality and relevance of the images obtained, leading to more accurate diagnoses. This approach aligns with the broader trend toward personalized medicine, where treatments and diagnostics are increasingly being tailored to the unique characteristics of each patient.

### 9.3. Expansion to Other Modalities

While AI-assisted low-dose protocols have seen the most significant developments in CT and X-ray imaging, there is substantial potential for expanding these technologies to other imaging modalities; fluoroscopy, positron emission tomography (PET), and nuclear medicine, which they also involve the use of ionizing radiation, could greatly benefit from AI-driven dose reduction strategies. Fluoroscopy, which is commonly used for real-time imaging during interventional procedures, often exposes patients to relatively high doses of radiation, especially in complex or lengthy procedures. By integrating AI into fluoroscopic imaging, it would be possible to optimize radiation doses in real-time based on the procedure’s requirements and the patient’s specific characteristics [151]. AI could also enhance the quality of fluoroscopic images by reducing noise and improving contrast, ensuring that clinicians can visualize critical structures while minimizing radiation exposure.

Similarly, a study agreed that PET and nuclear medicine, which involve the injection of radiotracers, could benefit from AI-assisted protocols [152]. AI could potentially optimize the amount of radiotracer administered, as well as enhance image reconstruction to reduce the need for higher doses [153]. By applying AI to these modalities, clinicians could achieve clearer, more accurate images with less radiotracer or radiation exposure, leading to safer imaging practices. The expansion of AI-assisted low-dose protocols to these modalities would mark a significant advancement in the field of medical imaging, ensuring that AI’s benefits in reducing radiation exposure and enhancing image quality extend across a broader range of diagnostic tools. This expansion could also improve the safety of imaging in vulnerable populations, such as children and individuals undergoing frequent follow-up studies, by reducing their cumulative radiation dose from multiple imaging modalities over time [154].

## 10. Challenges and Limitations of AI in Low-Dose Imaging

While AI-assisted low-dose imaging presents significant advancements in reducing radiation exposure and enhancing diagnostic quality, several challenges and limitations must be addressed for widespread clinical adoption. These challenges include issues related to model generalizability, ethical considerations, computational demands, and practical implementation in resource-constrained healthcare settings [47]. Understanding and overcoming these barriers is crucial to ensuring the broad applicability and equitable use of AI-driven low-dose imaging technologies.

### 10.1. Generalizability of AI Models

One of the key limitations in implementing AI in medical imaging is the challenge of generalizability across diverse patient demographics, imaging devices, and clinical settings. AI models are often trained on specific datasets collected from particular populations and imaging systems, which may not fully represent the variability in real-world clinical practice. Differences in patient anatomy, imaging protocols, manufacturer-specific scanner settings, and institutional workflow variations can impact AI performance when applied outside its training domain. Studies have shown that AI models trained on data from high-income countries may exhibit reduced performance when tested on datasets from underrepresented regions with different population characteristics [155,156]. Additionally, AI systems may struggle with rare diseases or uncommon imaging artifacts not well-represented in the training data, leading to potential misdiagnoses (Sanida). To improve generalizability, efforts should focus on multi-institutional collaborations, dataset diversification, and model validation across multiple geographic and clinical contexts.

### 10.2. Ethical and Regulatory Considerations

Ethical challenges surrounding AI in medical imaging remain a significant concern, particularly regarding algorithmic bias, data privacy, and equitable access to AI-driven technologies [157]. Algorithmic bias arises when AI models inherit disparities present in the training data, potentially leading to inaccurate predictions for certain demographic groups. For instance, studies have indicated that AI-based diagnostic models may demonstrate variations in sensitivity and specificity when applied to different racial, ethnic, or gender groups, exacerbating healthcare disparities. Addressing this requires transparent model auditing, bias mitigation strategies, and diverse dataset representation to ensure fairness in AI decision-making.

Data privacy is another critical issue, as AI models require large volumes of medical imaging data for training. Ensuring compliance with privacy regulations such as the General Data Protection Regulation (GDPR) and the Health Insurance Portability and Accountability Act (HIPAA) is essential for maintaining patient confidentiality. The use of federated learning, where AI models are trained across multiple institutions without directly sharing patient data, has been proposed as a solution to mitigate privacy risks.

Additionally, disparities in access to AI-driven imaging technologies create inequities between well-resourced and low-resource healthcare settings. AI models are often developed in high-income regions with access to state-of-the-art imaging infrastructure, while resource-limited hospitals may lack the necessary computational power or expertise to implement AI-driven imaging solutions effectively. Strategies to increase accessibility, provide regulatory guidelines for AI deployment, and develop AI models optimized for low-resource settings are essential for equitable healthcare integration.

### 10.3. Computational Demands and Practical Implementation

AI models, particularly deep learning architectures such as convolutional neural networks (CNNs) and generative adversarial networks (GANs), require substantial computational resources for both training and inference. The high-performance graphics processing units (GPUs) and cloud-based computing infrastructure necessary for AI-based medical imaging can be costly and inaccessible in many healthcare environments, especially in developing regions. Even in well-resourced settings, the integration of AI into clinical workflows requires significant software and hardware investments, as well as continuous model updates to maintain accuracy and relevance.

To address these challenges, efficient AI models with lower computational complexity should be developed for deployment in resource-limited settings. Techniques such as knowledge distillation, where a large AI model trains a smaller, more efficient model, and edge computing, which enables AI-driven image enhancement directly on imaging devices, could make AI-assisted low-dose imaging more feasible in diverse healthcare environments. Additionally, AI developers should prioritize hardware-agnostic algorithms that can run on standard hospital infrastructure rather than requiring expensive proprietary systems.

### 10.4. Open Challenges and Future Considerations

Despite its promise, AI-assisted low-dose imaging still faces several open challenges that require further investigation and development. Many AI models function as “black boxes”, making it difficult for radiologists to understand and validate AI-driven image enhancements. Developing explainable AI (XAI) frameworks that provide insights into how AI algorithms generate results is critical for clinical trust. Also, with regards to real-time AI Integration, while AI excels in post-processing, achieving real-time AI-driven optimization of radiation dose during imaging acquisition remains an area for further innovation. Furthermore, the lack of standardized guidelines for AI in medical imaging will delay its clinical adoption and regulatory approvals. Developing benchmarking standards and ensuring compliance with regulatory frameworks will be essential.

## 11. Conclusions

AI-assisted low-dose imaging represents a major advancement in medical imaging, addressing the critical challenge of reducing radiation exposure while maintaining high diagnostic accuracy. By leveraging advanced AI techniques such as noise reduction, artifact correction, and real-time optimization of imaging parameters, AI-driven solutions significantly enhance image quality at lower radiation doses. This is particularly beneficial for vulnerable populations, including pediatric patients, cancer patients, and individuals requiring frequent imaging. AI’s ability to improve diagnostic outcomes while minimizing radiation exposure marks a crucial step toward safer and more effective imaging practices, reducing the long-term risks associated with ionizing radiation.

Despite its transformative potential, the widespread adoption of AI-assisted low-dose imaging faces several challenges. Ensuring model generalizability across diverse patient populations, imaging devices, and clinical settings remains a key hurdle, as AI models must perform consistently across varying imaging environments. Additionally, regulatory frameworks must evolve to ensure AI’s safety, transparency, and fairness in clinical applications, addressing concerns related to algorithmic bias and ethical considerations. The computational demands of AI-based imaging solutions also pose challenges, particularly for smaller healthcare institutions with limited technological infrastructure. Overcoming these barriers will require interdisciplinary collaboration between AI researchers, radiologists, and policymakers to ensure responsible and effective AI integration into clinical workflows.

As AI technologies continue to evolve, they are poised to become an essential component of modern radiology, enhancing patient safety, workflow efficiency, and diagnostic precision. The future of radiology will likely see AI-driven imaging solutions becoming standard practice, enabling a more personalized, efficient, and safer approach to patient care. The integration of AI into medical imaging not only represents a technological evolution but a fundamental shift towards precision-driven and patient-centered healthcare.

## Figures and Tables

**Table 1:** Evaluation metrics for AI-based low-dose imaging techniques.

Metric	Description	Typical Use Case	Performance Range in AI Models
Structural Similarity Index (SSIM)	Measures structural similarity between AI-enhanced and full-dose images. SSIM values range from 0 to 1, with higher values indicating a closer resemblance to reference images [93].	Used in evaluating AI-based denoising and artifact reduction in CT and MRI [94].	AI-based low-dose imaging models often achieve SSIM scores of ≥0.90, indicating near-full-dose image quality [95].
Peak Signal-to-Noise Ratio (PSNR)	Evaluates the ratio between signal power and noise level in decibels (dB). Higher PSNR values indicate lower noise and better image clarity [96].	Applied in noise suppression and super-resolution image enhancement [97].	AI-driven reconstruction techniques typically improve PSNR by 4–6 dB over conventional low-dose methods [98].
Contrast-to-Noise Ratio (CNR)	Measures how well contrast is preserved while suppressing noise, which is critical for detecting lesions and fine anatomical details [99].	Used in AI-assisted contrast enhancement techniques [100].	AI-based methods enhance CNR by 20–30%, improving lesion detectability [101].
Mean Squared Error (MSE)	Quantifies pixel-level differences between AI-enhanced images and reference full-dose images. Lower MSE values indicate better reconstruction accuracy [93].	Used in evaluating AI-driven super-resolution and artifact removal methods [102].	AI-enhanced image processing techniques have shown 40% lower MSE compared to conventional approaches [103].
Root Mean Squared Error (RMSE)	A variation of MSE that gives higher weight to larger pixel deviations, providing a more comprehensive assessment of image accuracy [104].	Applied in evaluating AI-based image restoration methods [105].	RMSE values are significantly reduced in AI-optimized imaging, leading to more reliable reconstructions [106].
Area Under the Receiver Operating Characteristic Curve (AUC-ROC)	Assesses the ability of AI models to distinguish between normal and abnormal cases, often used in AI-based diagnostic classification [107].	Used in AI-assisted lesion detection in low-dose CT and MRI [108].	AI-driven detection models report AUC-ROC values of ≥0.95, demonstrating high diagnostic reliability [109].
Dice Similarity Coefficient (DSC)	Measures segmentation accuracy by comparing AI-identified regions to expert-annotated reference regions. Values closer to 1 indicate better segmentation [110].	Applied in AI-based segmentation tasks in CT and MRI [111].	AI-based segmentation models achieve DSC values ≥ 0.85, ensuring precise anatomical delineation [112].

**Table 2:** Key applications of AI in low-dose imaging protocols.

Imaging Modality	AI Technique	Function	Benefits	Example/Study
CT	CNNs	Denoising, artifact reduction, image reconstruction	Improve LDCT image quality by reducing noise, enhancing resolution, and maintaining diagnostic accuracy even at lower radiation doses	A study found that AI models effectively denoised LDCT images and improved visualization of lung nodules without increasing radiation exposure [74].
	GANs	Image synthesis and enhancement	Generate high-quality images by learning from full-dose counterparts, offering significant dose reductions without sacrificing diagnostic utility. GANs have been explored to augment the training data for distinction capabilities of disease detection models.	The use of GANs increased the detection rate of metastatic liver lesions in abdominal CT scans from 65% to 95% [113]. The use of GANs increased the anomaly score on malignant images from 91.6% to 95.32% [114]. A study found that the use of GANs for data augmentation improved the detection accuracy of sub-centimetric pulmonary adenocarcinoma by about 8%, increasing from 53.2% to 60.5% [115].
X-ray Imaging	ML Models	Denoising, contrast enhancement, feature extraction	Allows for lower radiation doses, especially in pediatric, emergency and dental settings, by reducing noise and improving anatomical visualization	A study demonstrated that the Quadratic SVM model achieved a detection accuracy of 97.58% for pneumonia in the pediatric age group [116]. An AI algorithm from TorchXRayVision achieved an accuracy of 76.54% when applied to a publicly available pediatric chest X-ray dataset [117].
	DL-based models	Segmentation, feature detection for abnormalities	Enhance the detection of subtle fractures, lung lesions, or infections with low-dose X-rays, improving speed and accuracy in diagnostic workflows	A study using a fuzzy enhanced deep learning-based framework to differentiate between chest X-rays of COVID-19 pneumonia and interstitial pneumonias not caused by COVID-19 achieved a classification accuracy of up to 81% [118].
MRI	AI-driven reconstruction algorithms	Motion artifact correction, noise reduction, acceleration of image acquisition	Reduce scan times and patient discomfort, improve image resolution, reduce the need for contrast agents in certain cases	The integration of advanced image processing algorithms and deep learning models reduced scan time by an average of 20–30%, resulting in quicker and more efficient experience [119]. AI-enhanced imaging techniques improved image quality at lower radiation doses, reducing the need for repeat scans and leading to a 25% reduction in repeat scan rates, directly enhancing patient experiences [120]. A study collected quantitative data from surveys and found that over 80% of patients reported a positive overall experience with the improved imaging procedures, which led to a 25% reduction in repeat scan rates, directly enhancing patient experiences [121].
	Reinforcement Learning	Optimization of scan parameters during acquisition	Adapts scan settings in real-time based on patient-specific factors, leading to more efficient scans and reduced need for operator intervention	A novel algorithm for breast lesion detection from DCE-MRI achieved optimal detection accuracy with reduced run time complexity [122].

Abbreviations—AI: artificial intelligence; CT: computed tomography (CT); CNNs: convolutional neural networks; LDCT: low-dose computed tomography; GANs: generative adversarial networks; ML: machine learning; SVM: support vector machines; DL: deep learning; COVID-19: coronavirus disease 2019; MRI: magnetic resonance imaging; DCE: dynamic contrast-enhanced.

## Data Availability

Not applicable.
